# Editorial: Cellular and Molecular Mechanisms of Immune Checkpoint Blockers in Anti-leukemia/Lymphoma Immune Therapy

**DOI:** 10.3389/fonc.2022.872300

**Published:** 2022-03-07

**Authors:** Mazdak Ganjalikhani-Hakemi, Ling Xu, Andrey A. Zamyatnin, Alexandr V. Bazhin

**Affiliations:** ^1^ Department of Immunology, Faculty of Medicine, Isfahan University of Medical Sciences, Isfahan, Iran; ^2^ Institute of Hematology, School of Medicine, Key Laboratory for Regenerative Medicine of Ministry of Education, Jinan University, Guangzhou, China; ^3^ Institute of Molecular Medicine, Sechenov First Moscow State Medical University, Moscow, Russia; ^4^ Belozersky Institute of Physico-Chemical Biology, Lomonosov Moscow State University, Moscow, Russia; ^5^ Department of Biotechnology, Sirius University of Science and Technology, Sochi, Russia; ^6^ Faculty of Health and Medical Sciences, University of Surrey, Guildford, United Kingdom; ^7^ Department of General, Visceral, and Transplant Surgery, Ludwig-Maximilians-University Munich, Munich, Germany; ^8^ German Cancer Consortium (DKTK), Partner Site Munich, Munich, Germany

**Keywords:** immune checkpoint (ICP), immune checkpoint blockers (ICBs), immune therapy, leukemia, lymphoma

In the last few decades, immunotherapy has emerged as the fourth pillar of hematologic malignancy therapy. As a new approach, one of the most effective immunotherapies is immune checkpoint blockade by immune checkpoint inhibitors (ICIs), such as programmed death receptor-1 (PD-1), cytotoxic T lymphocyte-associated antigen-4 (CTLA-4) inhibitors (1, 2). Early-phase clinical trial results have demonstrated the remarkable effectiveness of ICIs in specific lymphoma subtypes, including classical Hodgkin lymphoma and primary mediastinal B-cell lymphoma. Unfortunately, ICIs have relatively disappointing effect in follicular lymphoma, diffuse large B-cell lymphoma and acute myeloid leukemia (AML), highlighting the role of cell-intrinsic and –extrinsic primary or acquired resistant mechanisms (3–5). Studies focus on identifying biomarkers to predict the efficacy of ICIs, seeking for other immune checkpoints (ICPs) with less adverse events and toxicity. Therefore, exploring for the cellular and molecular mechanisms of immune cell exhaustion is appreciated as a hot topic area of the hematologic malignancies research.

Early studies on the efficacy of ICIs suggest that the type of immune cells, PD-L1 expression, neoantigens, and genetic and epigenetic signatures can predict the response to immune checkpoint blockade and immune-related adverse events (irAEs). However, contradictory conclusions are obvious and hence, continually explore the highly reliable predictive biomarkers are still needed which will facilitate patient selection and decision-making related to immune checkpoint inhibitor-based therapies for leukemia and lymphoma patients (6, 7). Based on these, we announced this special issue by Frontiers in Oncology. The main purpose of the issue was to gather novel findings from scientists and clinicians involved in studying the cellular and molecular biomarkers which could predict the outcome of immune check-points blockers in treatment of leukemia and lymphoma.

Aside from the fact that check-point molecules play important roles in upholding the balance of immune function, patients treated with ICIs may suffer from a series of irAEs (8). Regarding the expensiveness of check-point blockade therapy (CBT), its application only for certain leukemia patients, and its potential toxicity or adverse effects, there is an urgent need for evaluation of biomarkers to identify and monitor the patient. In spite of availability of several potential biomarkers, further studies are needed since there is no consensus yet. Aru et al. tried to elucidate this situation and reviewed recent information on the predictive and prognostic value of biomarkers and laboratory methods for clinical follow-up of patients with hematologic malignancies who were treated with ICBs. Altogether, they believe that development of standardized methods for all of the present laboratory testings for CBT, working to offer cheaper and more accessible services to patients and development of new methods to be deliver in clinical laboratories are necessary.

Feng et al., in their case report, showed that the patient described finally achieved partial remission following the anti-CD19-CAR T cell plus PD-1 inhibitor therapy and subsequent maintenance therapy with PD-1 inhibitor. They believe that applying PD-1 inhibitor could improve the function of anti-CD19-CAR T cells by antagonizing the immunosuppressive microenvironment, while not increase the risk of acute rejection when combination with tacrolimus in the patients with refractory PTLD.

Newly discovered ICP receptors as well as their ligands or small molecular inhibitors for treatment of leukemia and lymphoma are another interesting field of research. Rezaei et al. put a great effort to present recent findings about TIM-3 as an attractive ICP in different types of hematologic malignancies. The authors stated that blockade of TIM-3 may end up to different outcomes in different leukemias and it is necessary to define how TIM-3 affects the function of immune cells, and if there are other links between TIM-3 overexpression and resistance to different therapies, relapse, and overall survival (OS). It is also asserted that blockade of TIM-3 alone fails to achieve clinical efficacy for most patients and its combination with other modalities could improves the clinical outcome.

Previous researches have demonstrated several cell-intrinsic and -extrinsic factors lead to the immune therapy resistance. For instance, transcription regulators, metabolic and epigenetic factors, immune suppressive soluble mediators and tumor metabolites in immune microenvironment, may lead to immune cell dysfunction before and after ICPs therapy (7, 9). Therefore, exploring the cell-intrinsic and -extrinsic mechanisms of the immune cell dysfunction in the course of immunotherapy may help to identify biological targets to conquer resistance to immunotherapy. Accordingly, an interesting study was received from Liang et al., who characterized the altered expression of thymocyte selection-associated HMG box (TOX) genes in AML and defined their different roles. They also demonstrated that the *TOX2* expression correlates positively with the expression of CTLA-4, PD-1, TIGIT, and PDL-2 genes, and are associated with poor OS for AML patients. This is probably due to the upregulation of these ICP genes. Based on their results, the authors concluded that TOX is also associated with the T cell exhaustion providing future direction for investigations of the possibility of TOX targeted inhibition by a suppressing proliferation of AML cells and restoring anti-AML T cell function.

In spite of a recent progression in the treatment of hematologic malignancies and the emergence of newer and more sophisticated therapeutic approaches such as immunotherapy, long-term OS is still unsatisfactory. Metabolic alterations are considered as an important cell-intrinsic hallmark of cancer cells, and do not only contribute to the malignant transformation of cells, but do also promote tumor progression and invasion. This point was thoughtfully discussed by Soltani et al.; their review summarize alterations in metabolic events providing cell requirements for rapid proliferation, drug resistance, self-renewal, etc. during leukemogenesis. Furthermore, the importance of cross-talk between immune and leukemia cells has been highlighted in their review, especially under complicated bone marrow microenvironment conditions. The authors asserted that although targeting metabolic molecules was entailed in clinical benefits in patients with leukemia, the effectiveness is still unsatisfactory and cytotoxic side effects are still unignorable. Thus, exploring metabolic requirements of leukemia cells to identify potential biomarkers with limited toxicities toward normal cells will be the future research focus.

At the whole, we received and reviewed with enthusiasm several interesting reviews and original papers for this issue which shed light into new research directions related to one of the most important and multi-disciplinary Research Topics - cellular and molecular mechanisms of immune checkpoint blockers in anti-leukemia/lymphoma immune therapy. We hope all of our efforts and the presented papers in this special issue could be interesting, informative and insightful for all of our readers to encourage them to take a close look into a subject of this collection.

## Author Contributions

All authors had contribution in writing, revising, and finalizing the manuscript.

## Conflict of Interest

The authors declare that the research was conducted in the absence of any commercial or financial relationships that could be construed as a potential conflict of interest.

## Publisher’s Note

All claims expressed in this article are solely those of the authors and do not necessarily represent those of their affiliated organizations, or those of the publisher, the editors and the reviewers. Any product that may be evaluated in this article, or claim that may be made by its manufacturer, is not guaranteed or endorsed by the publisher.
